# Indigenous Tocopherol Improves Tolerance of Oilseed Rape to Cadmium Stress

**DOI:** 10.3389/fpls.2020.547133

**Published:** 2020-10-23

**Authors:** Essa Ali, Zeshan Hassan, Muhammad Irfan, Shabir Hussain, Haseeb-ur- Rehman, Jawad Munawar Shah, Ahmad Naeem Shahzad, Murtaza Ali, Saad Alkahtani, Mohamed M. Abdel-Daim, Syed Asad Hussain Bukhari, Shafaqat Ali

**Affiliations:** ^1^Department of Food Science and Technology, Zhejiang University of Technology, Hangzhou, China; ^2^College of Agriculture, Bahauddin Zakariya University, Layyah, Pakistan; ^3^Department of Agronomy, Bahauddin Zakariya University, Multan, Pakistan; ^4^Department of Basic Sciences and Humanities, University of Engineering and Technology, Mardan, Pakistan; ^5^Department of Zoology, College of Science, King Saud University, Riyadh, Saudi Arabia; ^6^Department of Pharmacology and Toxicology, Faculty of Veterinary Medicine, Suez Canal University, Ismailia, Egypt; ^7^Department of Environmental Sciences and Engineering, Government College University, Faisalabad, Pakistan; ^8^Department of Biological Science and Technology, China Medical University, Taichung, Taiwan

**Keywords:** cadmium stress, oilseed rape, pigments, ultrastructure, tocopherols

## Abstract

Two oilseed rape genotypes (Jiu-Er-13XI and Zheyou-50), differing in seed oil content, were subjected to cadmium (Cd) stress in hydroponic experiment. Genotypic differences were observed in terms of tolerance to Cd exposure. Cd treatment negatively affected both genotypes, but the effects were more devastating in Jiu-Er-13XI (low seed oil content) than in Zheyou-50 (high seed oil content). Jiu-Er-13XI accumulated more reactive oxygen species (ROS), which destroyed chloroplast structure and decreased photosynthetic pigments, than Zheyou-50. Total fatty acids, especially 18:2 and 18:3, severely decreased as suggested by increase in MDA content. Roots and shoots of Jiu-Er-13XI plants accumulated more Cd content, while less amount of tocopherol (Toc) was observed under Cd stress, than Zheyou-50. Conversely, Zheyou-50 was less affected by Cd stress than its counterpart. It accumulated comparatively less amount of Cd in roots and shoots, along with reduced accumulation of malondialdehyde (MDA) and ROS under Cd stress, than Jiu-Er-13XI. Further, the level of Toc, especially α-Tocopherol, was much higher in Zheyou-50 than in Jiu-Er-13XI, which was also supported by high expression of Toc biosynthesis genes in Zheyou-50 during early hours. Toc not only restricted the absorption of Cd by roots and its translocation to shoot but also scavenged the ROS generated during oxidative stresses. The low level of MDA shows that polyunsaturated fatty acids in chloroplast membranes remained intact. In the present study the tolerance of Zheyou-50 to Cd stress, over Jiu-Er-13XI, is attributed to the activities of Toc. This study shows that plants with high seed oil content are tolerant to Cd stress due to high production of Toc.

## Introduction

Heavy metals are major environmental pollutants due to their detrimental effects on life. Severe concerns, related to ecology, evolution, nutrition, and environment, are associated with heavy metals (Hawkes, 1997). Due to exorbitantly increasing industrialization and urbanization, heavy metals are constantly penetrating the environment as industrial effluents and household wastes, posing a potential threat to the ecosystem. Heavy metals enter the food chain through plants grown on polluted soil or irrigated with contaminated water (Bukhari et al., 2016). Cadmium (Cd) is one of such hazardous metals piercing the environment by anthropogenic, as well as natural means (Lima et al., 2006). Cadmium becomes a part of the soil–plant environment as plants absorb water and nutrients from the soil. Cadmium can affect plants in many ways, especially by damaging their photosynthetic apparatus (Ali et al., 2015), reducing gas exchange ability (Jia et al., 2011), causing nutrient imbalance (Feng et al., 2013), subcellular changes like chloroplast, mitochondrial, and nuclear membrane disintegration (Liu et al., 1995; Wang et al., 2011; Ali et al., 2018), and destroying antioxidant defense systems (Ahmad et al., 2011). Moreover, exposure of plants to Cd stress inhibits cell growth (Di Toppi and Gabbrielli, 1999) and alters root morphology (Jia et al., 2011). In short, contamination of soil and water bodies with Cd and its subsequent absorption by plants are serious concerns threatening the agricultural production and human health globally (Wu et al., 2005). Cd-induced damage to plants is an outcome of overgeneration of reactive oxygen species (ROS). Cadmium cannot directly generate free radicals via Fenton and/or Haber Weiss reactions in biological systems. However, the accumulation of ROS in Cd-treated plants has been reported in literature (Pathak and Khandelwal, 2006; Zhou et al., 2009). Actually, Cd produces ROS indirectly by increasing the concentration of free Fe by replacing it in different proteins, compromising the integrity of the membranes by lipid peroxidation, which can be demonstrated by the increase in malondialdehyde (MDA) content (Scholz et al., 1990; Dorta et al., 2003). Plants combat Cd stress by virtue of certain enzymatic and non-enzymatic systems. In plants, tocopherols are considered as an important component of non-enzymatic protection against stresses.

Oilseed rape (*Brassica napus* L.), an important crop of temperate regions, provides edible oil, animal feed, and biodiesel. Its dry seed contains about 40–50% oil content (Hüsken et al., 2005). Besides, oilseed rape contains lipid soluble tocopherols, also called vitamin E, which is a well-known antioxidant against reactive oxygen species (ROS), and an essential nutrient for human beings and animals. Tocopherols, the constituents of compounds having vitamin E, are synthesized by photosynthetic organisms only (Mène-Saffrané and DellaPenna, 2010). Tocopherols are lipid-soluble molecules with four structural derivatives [alpha (α), beta (β), gamma (γ), and delta (δ)] having diverse biological functions. Tocopherols serve as antioxidants by annihilating singlet oxygen and neutralizing the harmful radicals to inhibit lipid peroxidation of membranes (Sattler et al., 2004a; Krieger-Liszkay, 2005). *In vitro*, each molecule of α-, β-, γ-, and δ-tocopherol can protect up to 220, 120, 100, and 30 molecules of polyunsaturated fatty acids (PUFAs), respectively (Fukuzawa et al., 1982). Plant tissues vary massively in their tocopherol content and composition (Grusak et al., 1999). Photosynthetic tissues contain 10 to 50 mg tocopherol/g fresh weight (FW), with α- tocopherol the dominant one, while seeds vary from 300 to 2,000 mg tocopherol/g oil, where γ-tocopherol is a main constituent (Grusak, 1999). This variation in tocopherol content and composition is attributed to a variety of environmental and genetic factors. Photosynthetic tissues, especially leaves, are highly responsive to environmental variations. However, tolerant plants generally exhibit enhanced tocopherol levels, while susceptible ones display net reduction in tocopherol content under stress. Alpha tocopherol shows a remarkable increase under water deficit conditions (Garcia-Plazaola and Becerril, 2000; Munne-Bosch and Alegre, 2001). Further, tocopherol content of plants increase in response to various abiotic stresses like salinity, light, cold, and heavy metals, which may offer a supplementary line of defense against oxidative deterioration (Havaux et al., 2000; Munne-Bosch and Alegre, 2002b; Tang et al., 2016; Gramegna et al., 2019; Nowicka et al., 2020). Based on increasing tocopherol content in response to abiotic stresses (Kim et al., 2019), it is believed that protection of PUFAs from oxidative damage is the main function of tocopherols (Havaux et al., 2000; Collakova and DellaPenna, 2003; Abbasi et al., 2007; Collin et al., 2008). The current study was designed to probe the response of two *Brassica napus* genotypes, differing in oil content, to Cd stress in terms of fatty acids and tocopherol levels.

## Materials and Methods

### Plant Materials and Treatments

A hydroponic trial was carried out under natural light conditions in a net house of the Zijingang campus, Zhejiang University, Hangzhou, China. Two rapeseed genotypes, Zheyou-50 (high seed oil content) and Jiu-Er-13XI (low seed oil content), were used in the experiment (Du et al., 2015). The seeds were sown in trays containing 50% compost, 25% vermiculite, and 25% sand in the growth chamber. After 1 month, seedlings were transferred to the wire house to adapt to the outside environment. Morphologically similar and healthy seedlings were shifted to 5-L buckets having 4.5 L of nutrient solution (mg L^– 1^): (NH_4_)_2_SO_4_, 48.2; MgSO_4_, 65.9; K_2_SO_4_, 15.9; KNO_3_, 18.5; Ca(NO_3_)_2_, 59.9; KH_2_PO_4_, 24.8; Fe-citrate, 7; MnCl_2_–4H_2_O, 0.9; ZnSO_4_–7H_2_O, 0.11; CuSO_4_–5H_2_O, 0.04; HBO_3_, 2.9; H_2_MoO_4_, 0.01. The pH of the nutrient solution was maintained at 5.8 ± 0.1 using HCl or NaOH. The containers were covered with plates having eight evenly spaced holes (one seedling per hole) and placed in a net house. One week after transplantation, Cd (as CdCl_2_.2.5H_2_O) was added to the corresponding containers to form two treatments: basal nutrient solution (control), and 100 μM Cd (Cd). The containers were continuously aerated using pumps, and the nutrient solution was renewed after every 5 days. The experimental treatments were adjusted in randomized complete block design (RCBD) with three replications. Samples for gene expression and quantification of ROS were collected at 12, 24, and 48 h after treatment (HAT). For other parameters, samples were harvested at 3, 6, and 9 days after treatment (DAT) and stored at −80°C for subsequent analysis.

### Quantifying Leaf Photosynthetic Pigments

Photosynthetic pigment contents (Chl a, Chl b, and carotenoids) were determined according to the method described by Wang et al. (2009). Briefly, 10 fresh leaf disks (diameter, 1 cm) were placed in a glass tube containing a mixture of ethanol, acetone, and distilled water (4.5:4.5:1) and stored at 4°C for 48 h in the dark. The absorbance of the extract was noted at 645 and 663 nm on a spectrophotometer.

Formula for chlorophyll determination (mg/g FW):

Chl. (a) = 12.71 × OD663 − 2.59 × OD645/100
Chl. (b) = 22.88 × OD645 − 4.67 × OD663/100
Total chlorophyll = Chl. (a) + Chl. (b)

### Determination of MDA Content

Leaf tissues (0.5 g) were crushed and blended with 8 ml of 50 mM phosphate buffer (PBS, pH 7.8) solution under ice-cold conditions. Homogenate was subjected to centrifugation at 12,000 rpm and 4°C for 15 min. The supernatant was collected to determine malondialdehyde (MDA) content as described by Hodges et al. (1999). The mixture of reaction solution (5% TCA solution with 2.5 g TBA) and sample extract was heated at 95°C for 15 min in a water bath, followed by immediate cooling on ice. After centrifugation at 4,800 rpm for 10 min, the supernatant was run on a spectrophotometer to record the absorbance at 532 nm (E532 = 1.55 × 10^–1^ mM cm^–1^). Non-specific turbidity was corrected by subtracting the value of absorbance taken at 600 nm.

### Determination of Cd Accumulation

About 0.1 g of oven-dried (at 65–70°C for 72 h) tissues of shoot and root were crushed for the determination of Cd concentration. Mineralization was performed by heating 5 ml of 65% HNO_3_ using a microwave digester (Microwave 3000; AntoonPaar). The volume of the digested samples was raised to 10 ml by adding Milli-Q water. Specimens were run on inductively coupled plasma-optical emission spectrometer (ICP-OES; Optima 8000DV; PerkinElmer) to quantify the Cd content.

### Leaf Tissue Reactive Oxygen Species Staining and Quantification

Leaves harvested at 12, 24, and 48 h after treatment were processed for ROS quantification. H_2_O_2_ and O_2_^–^ contents were quantified using the method suggested by Jiang and Zhang (2001) and Gong et al. (2008), respectively.

### Determination of Tocopherol Content

Tocopherols were extracted and analyzed by the method suggested by Li et al. (2013) with minor changes. About 100 mg of fresh leaf discs were placed in a 2-ml skirted screw-cap microtube having 1.3 ml of n-hexane. Samples were macerated using bead mill homogenizer (Bead Ruptor-24, Omni, Kennesaw, GA, United States) at 8 m s^–1^ for 30 s and incubated for 15 h under dark conditions. The supernatant (20 μl) was subjected to normal-phase high-performance liquid chromatography machine (model 600, Waters, Milford, MA, United States) fitted with a Zorbax Rx-SIL column (4.6 mm × 250 mm × 5 μm; Agilent, Englewood, CO, United States) and a fluorescence detector (λex = 295 nm; λem = 330 nm). The mobile phase of hexane/tert-butyl methyl ether (95:5, v/v) was transported at a constant rate of 1 ml min^–1^. Isoforms of tocopherol were quantified using the curves derived from pure standards of tocopherol. The peaks of the tocopherol standards (α-, β, γ-, and δ- tocopherol) were distinguished by their retention times. To determine tocopherol contents of experimental samples, standard curve was calibrated in accordance with the corresponding peaks of individual tocopherol derivatives. Tocopherol contents were expressed in mg per g of sample, and the total amount of tocopherol was calculated as the sum of α- and γ-tocopherol.

### Determination of Fatty Acid Composition and Content

Fatty acids (FAs) were analyzed according to the protocol described by Chen et al. (2012) with slight modifications. Approximately 200 mg of leaf samples were ground in a 12-mL screw-top glass tube having 5 ml of extraction solution (chloroform/isopropanol, 2:1, v/v), followed by incubation at 80°C for 2 h. Upon cooling down the samples to room temperature, 1 ml of n-hexane and 2 ml of NaCl solution (0.9%) were added to the tubes. The specimens were centrifuged at 2,300 rpm for 5 min. The supernatant (1 ml) was used to analyze the FA components on GC-FID. The samples (2 μl) were auto-injected into the gas chromatograph (GC, 6890N, Agilent, United States) system, having a fused silica capillary column Rtx-Wax (30 m × 0.25 mm × 0.50 μm, Restek, United States) and FID detector. Initially, the column temperature was set to 160°C for 1 min, following a gradual increase to 240°C at the rate of 4°C/min, and maintained for 16 min.

### Quantitative Real-Time PCR Analyses

About 100 mg of leaf tissues were crushed, and total RNA was extracted using Miniprep kit, following the manufacturer’s instructions. RNeasy Mini Kit (Qiagen, Hilden, Germany) was used to remove genomic DNA. ReverTra AceqPCR RT kit (Toyobo, Japan) was utilized to synthesize cDNA. Based on mRNA or expressed sequence tag (EST), gene-specific primers were designed for qRT-PCR. SYBR Green PCR Master Mix (Applied Biosystems) was used to amplify the PCR products, in triplicate, in 25 μl qRT-PCRs in an iCycleriQ^TM^ 96-well real-time PCR detection system (Bio-Rad, Hercules, CA, United States). PCR conditions were as follows: denaturation at 95°C for 3 min; 40 cycles of denaturation at 95°C for 30 s; annealing at 58°C for 30 s; extension at 72°C for 30 s. Threshold cycle values were calculated by employing the software provided with the PCR machine and mRNA levels were quantified by following the method described by Livak and Schmittgen (2001).

### Subcellular Analysis by Transmission Electron Microscopy (TEM)

Fresh leaf sections (∼1 mm^2^) were excised at 9 days after treatment (DAT) from the topmost fully expanded leaves of plants and processed for TEM studies. Samples were fixed in 2.5% glutaraldehyde (v/v) overnight and washed three times with 0.1 M SPB (sodium phosphate buffer, pH 7.0). The specimens were post-fixed in 1% osmium tetroxide (OsO_4_) for 1 h and washed with 0.2 M SPB (pH 7.2) for 1–2 h. Dehydration was carried out in a graded series of ethanol (50, 60, 70, 80, 90, 95, and 100%) and acetone (100%). The samples were then infiltrated and embedded in Spurr’s resin. Ultrathin sections (80 nm) were prepared and mounted on copper grids and viewed under transmission electron microscope (JEOL TEM-1230EX) at an accelerating voltage of 60.0 kV.

### Statistical Analysis

Data were statistically analyzed using MSTAT-C software version 2.10 and expressed as means ± standard error (SE). Data were subjected to one-way ANOVA, and LSD test was used to compare the treatments at *P* ≤ 0.01 or *P* ≤ 0.05 (Steel and Torrie, 1980). Figures were prepared using OriginPro v7.5 (OriginLab, Northampton, MA, United States) software.

## Results

### Photosynthetic Pigment Composition

Chlorophyll is the main component of green plants. The amount and composition of chlorophyll differ from genotype to genotype and also depend on environmental conditions. In the present study, total chlorophyll content of Zheyou-50 was 52.3 mg g^–1^ FW, which was considerably higher than that of Jiu-Er-13XI (38.1 mg g^–1^ FW). Chl a and Chl b contents also varied between two genotypes. Zheyou-50 and Jiu-Er-13XI contained 32.1 and 23.5 mg g^–1^ FW chl a, and 21.1 and 14.6 mg g^–1^ FW chl b, respectively (Table 1). Cd treatment adversely affected pigment contents in both genotypes. Chl a, chl b, and total chl decreased by 32.35, 21.27, and 30% respectively, with respect to control treatment. The detrimental effect of Cd became prominent with increasing treatment time duration. Total chlorophyll content reduced by 10 and 20% at 6 DAT and 9 DAT, respectively, compared to 3 DAT (Table 1). All sole factors were significantly different for photosynthetic pigment composition and content.

**TABLE 1:** Sole effect of genotype, treatment, and timings on photosynthetic pigments in rapeseed.

	Chl a [mg/g fresh weight (FW)]	Chl b (mg/g FW)	Chla + b (mg/g FW)
**Genotypes**			
Jiu-Er-13XI	23.5 ± 2.07 b	14.6 ± 0.99 b	38.1 ± 2.95 b
Zheyou-50	32.1 ± 1.15 a	21.1 ± 0.88 a	52.3 ± 2.03 a
Significance	**	**	**
**Treatments**			
Ck	33.2 ± 1.08 a	20.0 ± 0.85 a	53.2 ± 1.79 a
Cd	22.4 ± 1.79 b (−32.35%)	15.7 ± 1.32 b (−21.27%)	37.2 ± 2.83 b (−30%)
Significance	**	**	**
**Timings**			
3DAT	31.1 ± 2.10 a	19.3 ± 1.31	50.4 ± 3.19 a
6DAT	27.1 ± 2.54 b (−12%)	17.9 ± 1.47 (−7.33%)	45.0 ± 3.72 ab (−10.7%)
9DAT	25.2 ± 2.35 b (−18%)	16.4 ± 1.66 (−14.84%)	40.1 ± 3.83 b (−20.36%)
Significance	**	ns	**

DAT, days after treatment; ns, non-significant. **Significant at *P* ≤ 0.01; numbers in parenthesis show percent increase (+) or decrease (−).

**FIGURE 1:**
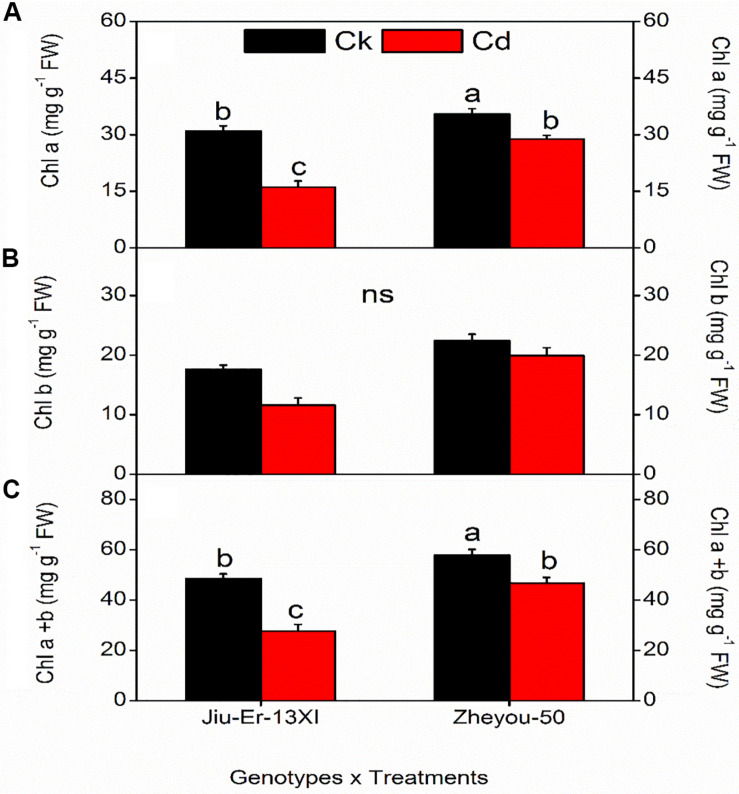
Interactive effect of genotype and treatment on photosynthetic pigments in rapeseed. Lettering indicates statistical difference (*p* ≤ 0.01) among the treatments for each parameter. Data represent the mean ± SE of three measurements. Ck, Cd, and ns represent control, 100 μM Cd, and non-significant, respectively.

Interactive effect showed a conspicuous decline in chlorophyll content in both genotypes subjected to Cd stress. However, the decrease was more pronounced in Jiu-Er-13XI than in Zheyou-50. Jiu-Er-13XI showed 47.9, 34.10, and 43% reduction in chl a, chl b, and total chlorophyll, respectively, compared to control treatment (Figure 1). However, both genotypes showed a non-significant decrease in pigment content with increase in treatment timing (Supplementary Table S2).

### Cadmium Accumulation and MDA Content

**FIGURE 2:**
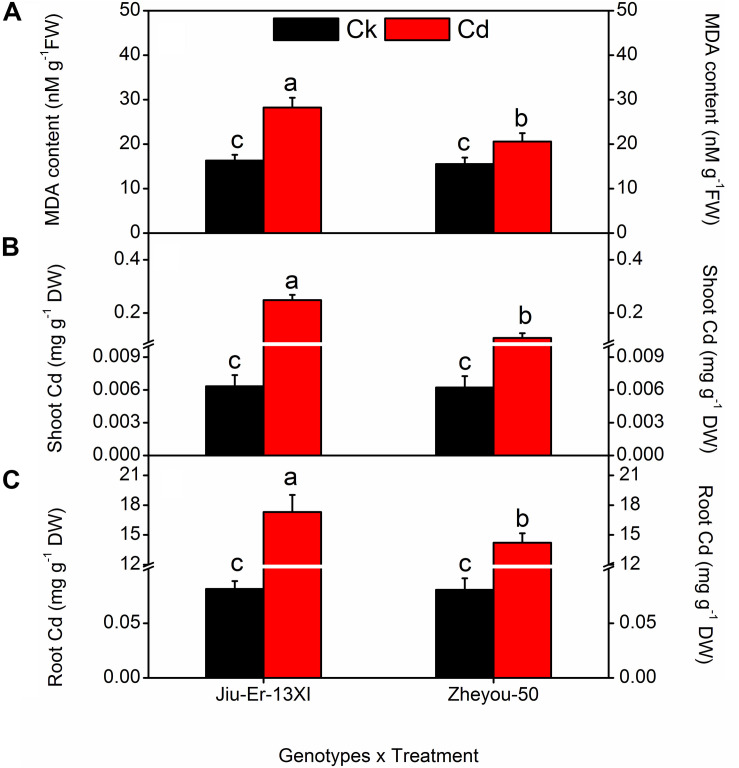
Interactive effects of genotypes and treatments on root, shoot, Cd accumulation, and leaf malondialdehyde (MDA) content in rapeseed. Lettering indicates statistical difference (*p* ≤ 0.01) among the treatments for each parameter. Data represent the mean ± SE of three measurements. Ck and Cd represent control and 100 μM Cd, respectively.

Malondialdehyde is the oxidative product of membrane lipids and its accumulation in plant tissues is an indicator of lipid peroxidation during environmental stresses. In the present study, Cd-treated plants accumulated up to 53% higher MDA content than the Cd-untreated ones, showing the deleterious effects of Cd on membrane lipids. Nonetheless, Zheyou-50 exhibited tolerance to Cd by accumulating less amount of MDA (18.72 nM g^–1^ FW) than Jiu-Er-13XI (22.27 nM g^–1^ FW). Maximum increase (63.15%) in MDA content was seen at 9 DAT, compared with 3 DAT, suggesting a rise in oxidative stress on membrane lipids with increasing time duration (Table 2). Interactive effects for MDA accumulation are presented in Supplementary Table S3 and Figure 2. Cd treatment deleteriously affected Jiu-Er-13XI and showed 72% higher MDA content than the control treatment. Conversely, Zheyou-50 depicted only 32% increase in MDA content under cd treatment compared to control. Moreover, 43.49, 58.63, and 46.93% increase was recorded for MDA accumulation at 3, 6, and 9 DAT, respectively, under Cd treatment. Roots are in direct contact with metals whether in the soil or in a hydroponic system. In the present study, root and shoot accumulated 15.7 and 0.17 mg Cd/g DW, respectively, under Cd treatment. Cd accumulation significantly increased with treatment timings as 9.9 mg/g DW (root) and 0.11 mg/g DW (shoot) Cd content was recorded at 9 DAT, which was considerably higher than that observed after 3 days of treatment (Table 2). Under Cd treatment, Jiu-Er-13XI plants accumulated Cd at the rate of 0.019 and 17.30 mg/g DW in shoot and root, respectively. However, Zheyou-50 showed only 0.017 and 14 mg/g DW Cd in shoot and root, under Cd treatment, respectively (Figure 2). Jiu-Er-13XI exhibited 89.80%, while Zheyou-50 showed only 48% rise in Cd accumulation at 9 DAT compared to 3 DAT.

**TABLE 2:** Sole effect of genotype, treatment and timings on root shoot cadmium (Cd) accumulation and leaf malondialdehyde (MDA) content in rapeseed.

**Factors**	**Cd (root) (mg/g DW)**	**Cd (shoot) (mg/g DW)**	**MDA (nM g^–1^ FW)**
**Genotypes**			
Jiu-Er-13XI	8.69 ± 2.24 a	0.12 ± 0.03 a	22.27 ± 1.89 a
Zheyou-50	7.14 ± 1.77 b	0.05 ± 0.01 b	18.05 ± 1.31 b
Significance	**	**	**
**Treatments**			
Ck	0.08 ± 0.006 b	0.007 ± 0.0007 b	15.92 ± 0.94 b
Cd	15.76 ± 1.02 a	0.17 ± 0.02 a	24.40 ± 1.68 a (+53.18%)
Significance	**	**	**
**Timings**			
3DAT	5.88 ± 1.78 c	0.05 ± 0.02 c	15.67 ± 1.15 c
6DAT	7.90 ± 2.39 b	0.09 ± 0.03 b	19.25 ± 1.81 b (+22.9%)
9DAT	9.97 ± 3.08 a	0.11 ± 0.03 a	25.56 ± 2.08 a (+63.15%)
Significance	**	**	**

DAT, days after treatment; ns, non-significant. **Significant at *P* ≤ 0.01.

### Accumulation of Reactive Oxygen Species

Reactive oxygen species (ROS), produced during oxidative stress, are harmful to plants. ROS, especially hydrogen peroxide (H_2_O_2_) and superoxide radicals (O_2_^•^), generated during stresses directly attack membrane lipids and compromise the integrity of the membrane system. In the current study, H_2_O_2_ and O_2_^•^ contents increased by 36.30 and 18.68%, respectively, under Cd stress compared to the control treatment. Increase in treatment duration elevated the accumulation of ROS. At 48 HAT, H_2_O_2_ and O_2_^•^ contents increased up to 32 and 14.20% compared to 12 HAT (Table 3).

**TABLE 3:** Sole effects of genotypes, treatments, and timings on leaf reactive oxygen species (ROS) accumulation in rapeseed.

	**H_2_O_2_ (μmol g^–1^ FW)**	**O_2_^–^ (nmol m^–1^ g^–1^ FW)**
**Genotypes**		
Jiu-Er-13XI	42.7 ± 2.7 a	61.1 ± 2.0 a
Zheyou-50	33.2 ± 1.2 b	54.6 ± 1.2 b
Significance	**	**
**Treatments**		
Ck	32.1 ± 1.1	52.9 ± 1.0 b
Cd	43.8 ± 2.5a (+36%)	62.8 ± 1.7a (+18%)
Significance	**	**
**Timings**		
12HAT	32.4 ± 1.7 b	53.1 ± 1.7 b
24HAT	38.5 ± 2.9a (+18%)	59.7 ± 1.9a (+12%)
48HAT	43.0 ± 3.4 a (+32%)	60.7 ± 2.6a (+14%)
Significance	**	**

HAT, hours after treatment. **Significant at *P* ≤ 0.01.

**FIGURE 3:**
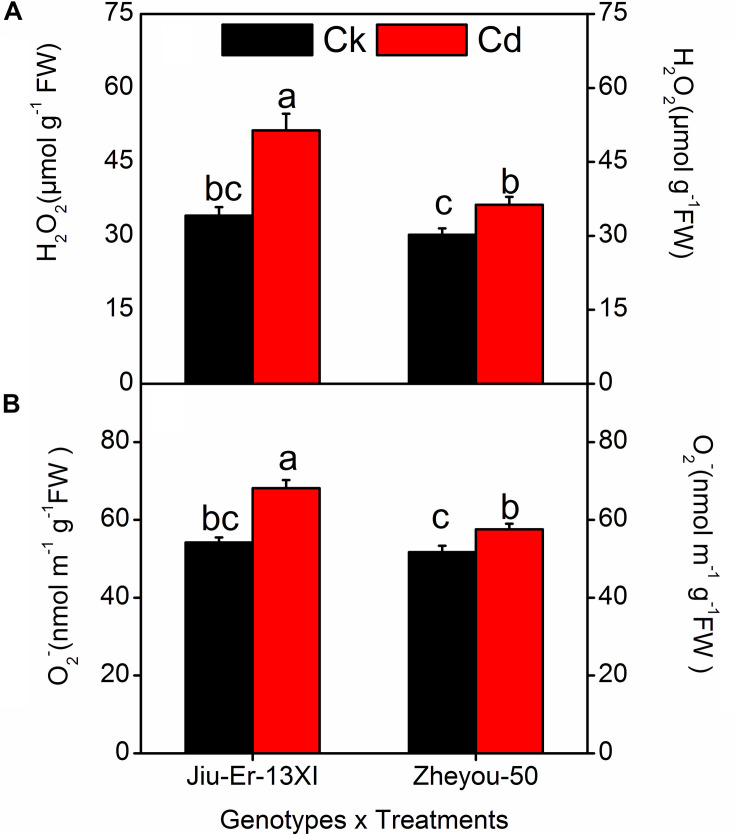
Interactive effects of genotypes and treatments on leaf reactive oxygen species (ROS) accumulation in rapeseed. Lettering indicates statistical difference (*p* ≤ 0.01) among the treatments for each parameter. Data represent the mean ± SE of three measurements. Ck and Cd represent control and 100 μM Cd, respectively.

Moreover, Jiu-Er-13XI was more severely affected by Cd stress than Zheyou-50. Jiu-Er-13XI showed 50 and 25.70%, while Zheou-50 exhibited only 20 and 11.32% increase in H_2_O_2_ and O_2_^•^ contents, respectively, in comparison with their respective control treatments (Figure 3). Treatment duration aggravated the outcomes of Cd stress on both genotypes. However, the impact was more pronounced in Jiu-Er-13XI, with a 43% increase in H_2_O_2_ content at 48 HAT, compared to 12 HAT (Supplementary Table S4).

### Expression of Tocopherol Biosynthesis Genes

**FIGURE 4:**
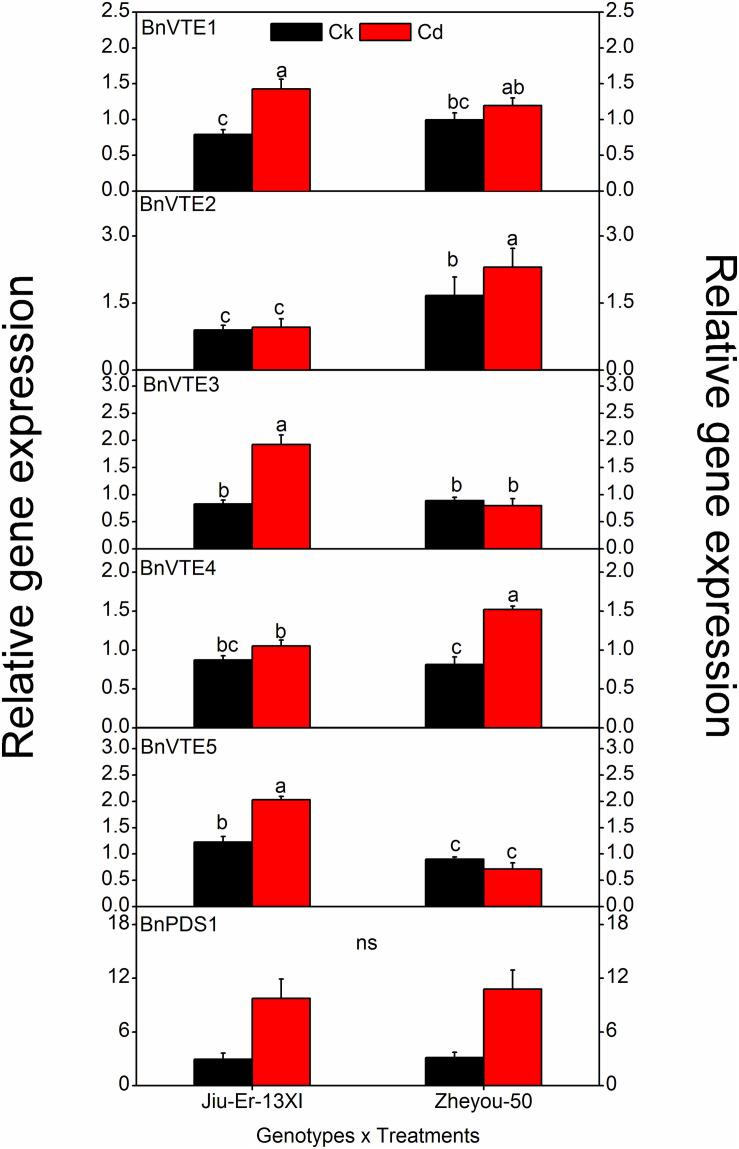
Interactive effects of genotypes and treatments on Toc genes expression in rapeseed. Lettering indicates statistical difference (*p* ≤ 0.01) among the treatments for each parameter. Data represent the mean ± SE of three measurements. Ck, Cd, and ns indicate control, 100 μM Cd, and non-significant, respectively.

Tocopherol biosynthesis pathway consists of six genes. Expression of these genes was measured at 12, 24, and 48 HAT. A significant difference was seen in the expression of all genes, except BnPDS1, in both genotypes. Moreover, Cd stress increased the expression of all genes compared to control treatment. An average increase of 52.7% was seen in BnVTE4, which codes for α-tocopherol. The increase in gene expression was time dependent (Table 4). Cadmium treatment affected the expression of tocopherol biosynthesis pathway genes in different manners in both genotypes. A prominent increase in BnVTE4 (87%) and BnVTE5 (65%) was observed in Zheyou-50 and Jiu-Er-13XI, respectively, under Cd stress, indicating the accumulation of α-tocopherol in Zheyou-50 and degradation of chlorophyll in Jiu-Er-13XI (Figure 4). Besides, a significant interaction was observed between genotypes and treatment as well as treatment and time duration except for BnVTE1 (Supplementary Table S5).

**TABLE 4:** Sole effects of genotypes, treatments, and timings on tocopherol (Toc) biosynthesis genes.

	**BnVTE1**	**BnVTE2**	**BnVTE3**	**BnVTE4**	**BnVTE5**	**BnPDS1**
**Genotypes**						
Jiu-Er-13XI	1.11 ± 0.11	0.93 ± 0.1 b	1.38 ± 0.16 a	0.96 ± 0.05 b	1.6 ± 0.1 a	6.4 ± 1.4
Zheyou-50	1.09 ± 0.08	1.98 ± 0.3 a	0.84 ± 0.07 b	1.17 ± 0.10 a	0.8 ± 0.06 b	7.0 ± 1.4
Significance	Ns	**	**	**	**	ns
**Treatments**						
Ck	0.89 ± 0.06 b	1.28 ± 0.23 b	0.86 ± 0.05 b	0.84 ± 0.05 b	1.06 ± 0.07b	3.0 ± 0.4b
Cd	1.31 ± 0.09 a	1.63 ± 0.28 a	1.36 ± 0.17 a	1.29 ± 0.07 a	1.37 ± 0.17a	10.3 ± 1.5a
Significance	**	**	**	**	**	**
**Timing**						
3HAT	1.15 ± 0.07	0.89 ± 0.08 b	1.11 ± 0.09	1.17 ± 0.07 a	1.17 ± 0.16	3.3 ± 1.1c
6HAT	1.09 ± 0.15	0.98 ± 0.17 b	1.08 ± 0.11	0.93 ± 0.1 ab	1.23 ± 0.13	5.7 ± 1.0b
9HAT	1.07 ± 0.11	2.50 ± 0.34 a	1.14 ± 0.27	1.09 ± 0.1b	1.26 ± 0.21	11.0 ± 0.02a
Significance	Ns	**	ns	**	Ns	**

HAT, hours after treatment; ns, non-significant. **Significant at *P* ≤ 0.01.

### Fatty Acid Contents and Composition

**FIGURE 5:**
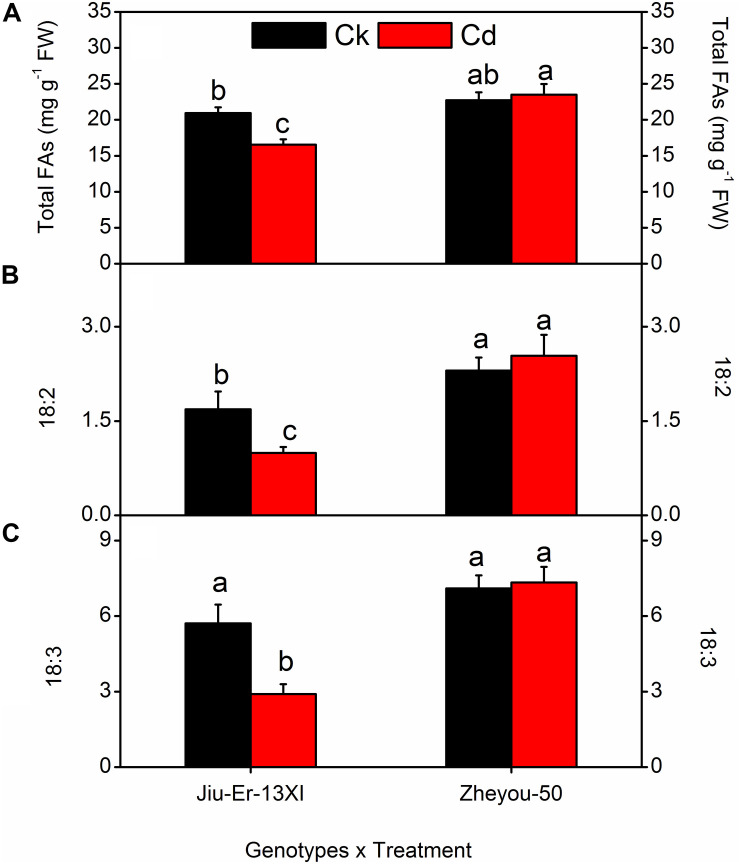
Interactive effects of genotypes and treatments on leaf fatty acid content and composition in rapeseed. Lettering indicates statistical difference (*p* ≤ 0.01) among the treatments for each parameter. Data represent the mean ± SE of three measurements. Ck and Cd represent control and 100 μM Cd, respectively.

Cadmium stress affected fatty acid contents and composition in a genotype-dependent manner. Compared with normal growth conditions, Jiu-Er-13XI showed about 21% decrease, while Zheyou-50 depicted 3.40% increase in total fatty acid content under Cd stress (Figure 5). Cd stress caused a reduction in 18:2 and 18:3 fatty acid contents of Jiu-Er-13XI by 41.11 and 49.21%, respectively, indicating a detrimental effect of Cd on polyunsaturated fatty acids (PUFA). In contrast, no such adversity was recorded for Zheyou-50. Instead, a 10.20 and 3.34% increase was detected in 18:2 and 18:3 fatty acids, respectively, suggesting a normal metabolism in this genotype. Sole and interactive effects are also presented in Table 5 and Supplementary Table S6.

**TABLE 5:** Sole effects of genotypes, treatments, and timings on leaf fatty acid (FA) content and composition in rapeseed.

	**16:0**	**18:0**	**18:1**	**18:2**	**18:3**	**Total FA**
**Genotypes**						
Jiu-Er-13XI	3.4 ± 0.09	1.39 ± 0.11	3.41 ± 0.18	1.34 ± 0.16	4.3	18.7 ± 0.74
Zheyou-50	3.3 ± 0.16	1.56 ± 0.07	2.85 ± 0.07	2.41 ± 0.19	7.2	23.1 ± 0.90
Significance	ns	ns	**	**	**	**
**Treatments**						
Ck	3.3 ± 0.14	1.33 ± 0.09	3.05 ± 0.15	1.99 ± 0.18	6.40	21.8 ± 0.69
Cd	3.5 ± 0.12 (+5%)	1.61 ± 0.08 (+21.4%)	3.21 ± 0.15 (+5.13%)	1.76 ± 0.25 (−11.5%)	5.11 (−21%)	20.0 ± 1.16 (−8.29%)
Significance	*	**	ns	**	**	**
**Timings**						
3DAT	2.9 ± 0.05	1.13 ± 0.13	3.48 ± 0.12	1.02 ± 0.12	4.72	17.6 ± 0.56
6DAT	3.3 ± 0.11 (+14.4%)	1.55 ± 0.07 (+37.2)	3.34 ± 0.19 (−3.9%)	2.12 ± 0.20 (107%)	5.49 (+16%)	21.2 ± 0.89 (+20.2%)
9DAT	4.0 ± 0.10 (+21.2%)	1.74 ± 0.06 (+12.3)	2.56 ± 0.10 (−26.5%)	2.4 ± 0.25 (141%)	7.06 (49%)	23.9 ± 1.24 (+12.9%)
Significance	**	**	**	**	**	**

DAT, days after treatment; ns, non-significant. *Significant at *P* ≤ 0.05; **significant at *P* ≤ 0.01.

### Tocopherol Contents and Composition

**FIGURE 6:**
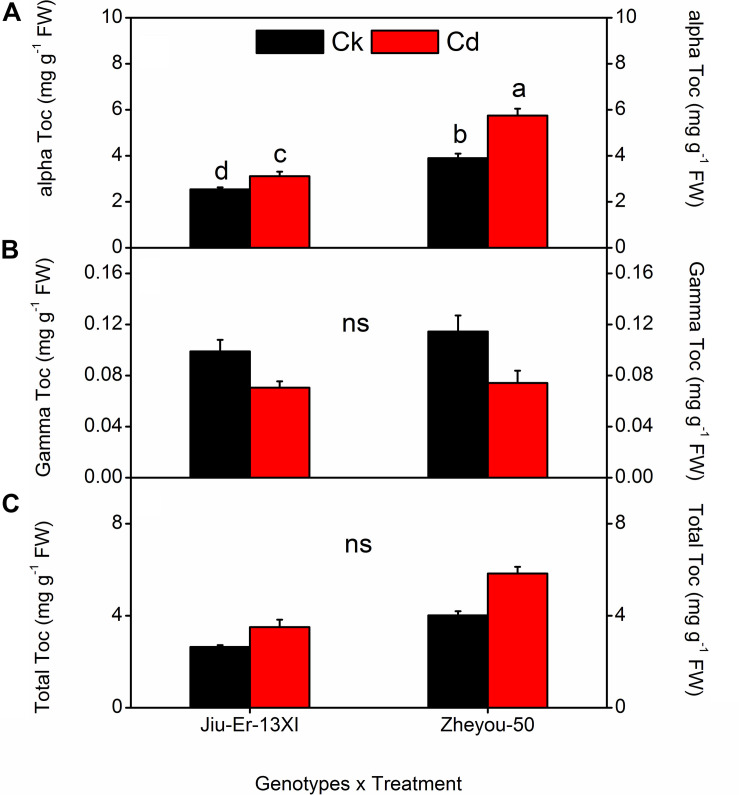
Interactive effects of genotypes and treatments on leaf tocopherol content and composition in rapeseed. Lettering indicates statistical difference (*p* ≤ 0.01) among the treatments for each parameter. Data represent the mean ± SE of three measurements. Ck, Cd, and ns represent control, 100 μM Cd, and non-significant, respectively.

Jiu-Er-13XI and Zheyou-50 showed a differential response to Cd in terms of tocopherol composition. Jiu-Er-13XI revealed 2.82, 0.08, and 3.07 mg g^–1^ FW of alpha, gamma, and total tocopherol, respectively, whereas Zheyou-50 exhibited a higher concentration of alpha (4.32 mg g^–1^ FW), gamma (0.09 mg g^–1^ FW) and total tocopherol (4.42 mg g^–1^ FW) than its counterpart. On average basis, Cd increased alpha and total tocopherol by 22.11 and 25.31%, respectively, while decreased gamma tocopherol content by 27.44% with respect to control. Moreover, treatment duration also enhanced the total tocopherol by 19.69 and 44.05% at 3 DAT and 9 DAT, respectively, with reference to the control treatment (Table 6). Considering interactive effects, Cd treatment increased total and α-tocopherol contents in both genotypes, while it decreased gamma tocopherol. However, the increase was prominent in Zheyou-50 as it accumulated 45.33 and 47.43% higher total and α-tocopherol under Cd stress than the control treatment. Conversely, Jiu-Er-13XI accumulated only 22.61 and 32.74% higher content of alpha and total tocopherol under Cd treatment than the normally grown plants (Figure 6). Treatment duration also induced the accumulation of α-tocopherol, which was higher (46.74%) in Zheyou-50 than in Jiu-Er-13XI (29.30%), with reference to their respective control treatments. The increase in total tocopherol was directly proportional to treatment duration. After 9 days of treatment, total and α-tocopherol increased by 41.86 and 36.20%, respectively. Interestingly, increase in α-tocopherol was accompanied by a corresponding decline in γ-tocopherol. Cd treatment decreased γ-tocopherol by 35.16 and 28.16% in Zheyou-50 and Jiu-Er-13XI, respectively. After 9 days of treatment, the decrease was considerably prominent in Zheyou-50 (51.08%), suggesting the impact of time duration on the fate of γ-tocopherol (Supplementary Table S7).

**TABLE 6:** Sole effects of genotypes, treatments, and timings on leaf tocopherol content and composition in rapeseed.

	**Alpha (mg g^–1^ FW)**	**Gamma (mg g^–1^ FW)**	**Total Toc (mg g^–1^ FW)**
**Genotypes**			
Jiu-Er-13XI	2.8 ± 0.1 b	0.08 ± 0.006b	3.0 ± 0.19 b
Zheyou-50	4.3 ± 0.2 a	0.09 ± 0.006a	4.4 ± 0.19 a
Significance	**	**	**
**Treatments**			
Ck	3.2 ± 0.1 b	0.10 ± 0.007 a	3.3 ± 0.19 b
Cd	3.9 ± 0.2 a (+22%)	0.07 ± 0.005 b (−27%)	4.1 ± 0.26 a (+25%)
Significance	**	**	**
**Timings**			
3DAT	2.9 ± 0.1c	0.11 ± 0.009 a	3.0 ± 0.18 c
6DAT	3.6 ± 0.2 b (+21%)	0.08 ± 0.006 b (−24%)	3.7 ± 0.25 b (+19.6%)
9DAT	4.1 ± 0.3 a (+14%)	0.06 ± 0.003 c (−43%)	4.4 ± 0.34 a (+44.0%)
Significance	**	**	**

DAT, days after treatment; ns, non-significant. **Significant at *P* ≤ 0.01.

### Subcellular Study

**FIGURE 7:**
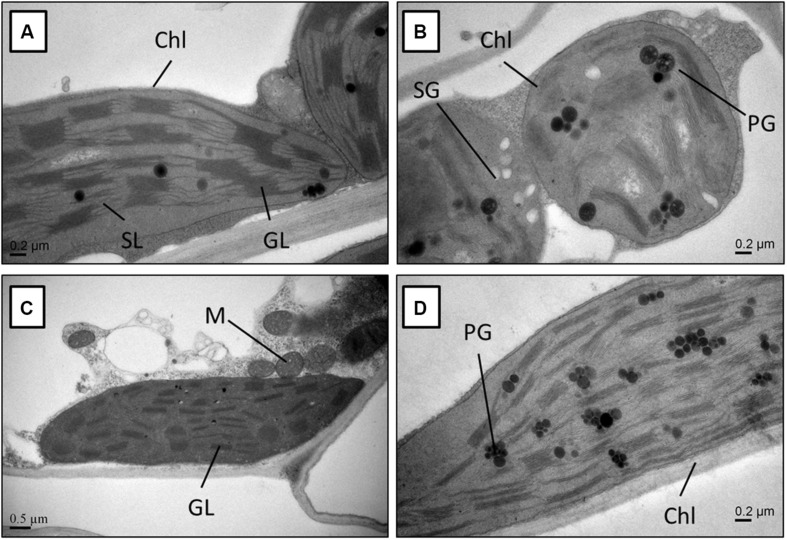
Transmission electron micrograph of the leaf mesophyll cells from Jiu-Er-13XI (A = Ck, B = 100 μM Cd), and Zheyou-50 (C = Ck, D = 100 μM Cd). Labels: Ch, chloroplast; SG, starch grain; PG, plastoglobule; M, mitochondrion; GL, granallamella; SL, stromal lamella.

Molecular and biochemical changes observed in rapeseed genotypes under Cd stress reflected at subcellular level as well. The leaf cells of the Jiu-Er-13XI and Zheyou-50 control plants were metabolically active, as suggested by the presence of well-developed chloroplasts with the normal shape and size. There were no starch granules in cells of both genotypes under control treatment, indicating a normal carbohydrate metabolism. Furthermore, the structure of granal and stromal lamella remained intact under normal growth environment (Figures 7A,C). Contrarily, subcellular structure of Cd-treated plants, especially Jiu-Er-13XI (low oil rapeseed genotype), was severely affected by stress. The chloroplast structure changed from normal elongated to big round-shaped one. The thylakoid membrane system was completely destroyed by Cd stress. Accumulation of starch granules in chloroplast is a clear sign of disrupted starch metabolism. Increase in number and size of plastoglobules suggests the accumulation of tocopherols under stress (Figures 7B,D). Unlike Jiu-Er-13XI, the genotype with high oil content (Zheyou-50) showed a moderate level of Cd-induced damage to chloroplast structure. The thylakoid membrane network showed comparatively better arrangements, with no starch granules, showing undisturbed carbohydrate metabolism under Cd stress.

## Discussion

Comprehensive study on tolerance of plants to Cd stress is of utmost importance to elucidate the underlying mechanisms to develop Cd-tolerant genotypes. Ability of plants to detoxify ROS is one of the main strategies to combat heavy metal-induced oxidative stress. Tocopherols are regarded as the non-enzymatic component of the plant defense system and play major roles in protecting plants against stresses at cellular and subcellular level (Collakova and DellaPenna, 2003; Li et al., 2010). The current investigation is focused on illuminating the role of tocopherols in response to oxidative stress, induced by Cd, in two oilseed rape genotypes, differing in fatty acid content.

Current study indicates Cd accumulation in roots and its translocation to upper parts of plants in both genotypes. However, the degree of absorption and translocation is higher in Jiu-Er-13XI (low seed oil content) than in Zheyou-50 (high seed oil content), suggesting the tolerance of Zheyou-50 to Cd stress. Usually, tolerant genotypes either inhibit the absorption of heavy metals via roots or restrict their translocation to the leaves. The effect of Cd on photosynthetic pigments is well documented (Ali et al., 2015). Cd is believed to replace metals in metal-containing enzymes, e.g., substituting Ca ion in PSII leads to the inhibition of water splitting systems. Damage to structure of chloroplast, reduction in chlorophyll synthesis and its deterioration are the major reasons for reduction in chlorophyll content (López-Millán et al., 2009). The net loss of chlorophyll was much higher in Jiu-Er-13XI than in Zheyou-50. The main reason behind this tolerance is the reduced absorption and restricted translocation of Cd ions in Zheyou-50.

Accumulation of ROS in Cd-treated plants has been reported in the literature (Pathak and Khandelwal, 2006; Zhou et al., 2009). Though Cd cannot directly generate free radicals, it produces ROS indirectly by increasing the free Fe-concentration, possibly via replacement in various proteins (Scholz et al., 1990; Dorta et al., 2003). Quantification of H_2_O_2_ and O_2_^–^ in this study indicates an immediate accumulation of ROS upon Cd exposure. The degree of accumulation is treatment duration and genotype dependent, as Zheyou-50 accumulated less ROS than Jiu-Er-13XI. Upregulation of BnVTE5 in Jiu-Er-13XI shows the increased degradation of chlorophyll by ROS.

Polyunsaturated fatty acids, such as linolenic acid and linoleic acid in membrane lipids are very sensitive to oxidation caused by Cd-induced ROS (Frankel, 2005). Current experiment reveals a decline in total fatty acid content of Cd-treated plants of Jiu-Er-13XI, compared to the normally grown plants. This reduction in total fatty acids is ascribed to decrease in 18:2 and 18:3 fatty acids in Cd-treated Jiu-Er-13XI plants. Stressed plants of Jiu-Er-13XI indicate increase in content of other fatty acids like, 16:0, 18:0, and 18:1, suggesting an inhibition in desaturation ability of enzymes. Contrary to that, no such effect on fatty acid composition of Zheyou-50 plants is observed. Instead, considerable enhancement in total fatty acid content under Cd treatment suggests the tolerance of this genotype to Cd stress. The increase in expression level of BnVTE4 in Zheyou-50 may protect PUFA under stressed environment. In a current investigation, decrease in 18:2 and 18:3 fatty acid contents of Jiu-Er-13XI is accompanied by the accumulation of MDA. MDA is the product of oxidation of 18:3 fatty acid, which is regarded as an important indicator of fatty acid oxidation under stress (Sattler et al., 2003). With increase in treatment duration, marked increase can be seen in MDA content. Conversely, MDA accumulation is low in Zheyou-50 because Cd stress had no such effects on the fatty acid composition of this genotype.

A study of anatomical disorders under a stressful environment is highly important to understand the tolerance mechanism of plants at subcellular level. Studies on barley (Wang et al., 2011) and tomato (Gratão et al., 2009) indicate that chloroplast is highly vulnerable to Cd stress. The present study reveals a severe damage to chloroplast structure of plants of Jiu-Er-13XI under Cd stress. The damaging effect can be explained as disruption in chloroplast structure (Borges et al., 2004), with completely deformed thylakoid membranes (Vijaranakul et al., 2001). Abnormal chloroplast, having reduced amounts of chlorophyll, is unlikely to possess surplus energy. Therefore, the presence of starch granules in Jiu-Er-13XI genotype indicates the incapability of chloroplasts to metabolize reserve carbohydrates (Uzunova and Popova, 2000), indicating the vulnerability of this genotype to Cd. In contrast, Zheyou-50 is not much affected by Cd stress.

Tocopherols are vitamin E group of lipid-soluble compounds having multifunctional properties. Tocopherols are believed to protect PUFA from ROS, produced during oxidative stress either by quenching superoxide radicals or by terminating the chain reaction (Sattler et al., 2004b; Krieger-Liszkay, 2005). Having said that tocopherols are produced by the oxygenic organisms and are an essential component of human nutrition, their function is not well investigated in plants. The reason behind the scarcity of literature could be the disappointing results obtained in certain studies where complete absence of tocopherol did not show any significant influence on plant growth and metabolism (Havaux et al., 2005; Maeda et al., 2005). Therefore, the role of tocopherol in mitigating oxidative damage remained unexplored. Instead, its role in other processes like transport of sugars, cellular signaling, and seed germination and dormancy are reported (Sattler et al., 2004b). However, with the advent of mutation and transgenic techniques in *Arabidopsis* and *Synechocystis* sp. PCC6803, study on functions of tocopherols became possible by eliminating (Schledz et al., 2001) or increasing the concentration of tocopherol (Collakova and DellaPenna, 2001; Savidge et al., 2002) or replacing them with biosynthetic intermediates (Porfirova et al., 2002; Sattler et al., 2003) to uncover the underlying mechanism in plants. Accumulation of tocopherol can be observed in response to multiple abiotic stresses (Havaux et al., 2000; Munne-Bosch and Alegre, 2002a). In the present study, the concentration of total and α-tocopherol improved under Cd stress compared to the control treatment, suggesting its role during oxidative stresses. The role of tocopherols in ameliorating Cd stress is attributed to the activity of α-tocopherol. Alpha tocopherol content increased in both genotypes under stress. However, the magnitude of increase was considerably lower in Jiu-Er-13XI than in Zheyou-50, strengthening the idea that sensitive plants show net loss of tocopherol during oxidative stresses (Munne-Bosch, 2005). The increase in α-tocopherol was accompanied by the decrease in γ-tocopherol, suggesting the upregulation of VTE4.

The role of α-tocopherol is well known in protecting PUFA from oxidative damage (Sattler et al., 2004a). In the current experiment, the decrease in total fatty acid content of Jiu-Er-13XI might be due to the low production of tocopherol, while in Zheyou-50, the increase in net fatty acid content was attributed to the protection provided by α-tocopherol. Alpha tocopherol provided protection to PUFA in Zheyou-50 by quenching the superoxide radicals, as ROS accumulation was reduced in this genotype compared to the Jiu-Er-13XI. MDA is the oxidative product of linolenic acid (Farmer 2007). In Jiu-Er-13XI, decrease in linolenic acid was accompanied by increase in MDA, showing the peroxidation of lipid membranes, while there was no such increase or decrease shown by Zheyou-50 for MDA and linolenic acid, respectively.

This view is further strengthened by the structure of chloroplast (Figure 7). In Jiu-Er-13XI, the chloroplast is completely deformed with destroyed thylakoid membranes and accumulation of starch granules, suggesting the oxidation of PUFA by ROS, and abnormality in sugar transport (Figure 7B). Abnormality in starch metabolism of Jiu-Er-13XI under Cd stress suggests the alternate function of tocopherol other than antioxidant (Maeda et al., 2006). The function of tocopherols in restricting the absorption of Cd and inhibiting its transport to upper parts of plant is not well documented. However, in the present study, reduction in absorption of Cd by roots and its limited translocation to aerial plant parts in Zheyou-50 might have been mediated by tocopherols.

## Conclusion

The current study concludes that genotypic differences were observed in Cd tolerance of two oilseed rape varieties. Zheyou-50, with higher tocopherol content, exhibited better tolerance to Cd stress than Jiu-Er-13XI in terms of all studied parameters. The reduction in absorption and translocation of Cd and scavenging of reactive oxygen species by tolerant genotypes indicate the involvement of tocopherol in Cd stress amelioration. The current study suggests that the plants having high tocopherol content can withstand Cd stress effectively. The outcomes of the present investigation will serve as a source of valuable information for the scientific fraternity engaged in developing Cd-tolerant oilseed rape genotypes. However, further studies must be conducted in order to explore the underlying mechanism of tolerance to Cd stress in plants.

## Data Availability Statement

All datasets for this study are included in the article/Supplementary Material.

## Author Contributions

EA conceived and oversaw the work. EA, JMS, and SAHB executed the trial. ZH, MI, and SH made the figures and tables. HR, ANS, and MA analyzed the data. EA, MA-D, and SAHB wrote the manuscript. SAk and SAi revised the manuscript. All authors have carefully read and approved the article.

## Conflict of Interest

The authors declare that the research was conducted in the absence of any commercial or financial relationships that could be construed as a potential conflict of interest. The handling editor declared a shared affiliation with one of the authors EA at the time of review.
