# Supplementary material for: Indigenous Tocopherol Improves Tolerance of Oilseed Rape to Cadmium Stress
Source: Front Plant Sci. 2020 Oct 23;11:547133. doi: 10.3389/fpls.2020.547133 (PMC7644955; doi:10.3389/fpls.2020.547133)
Supplement: Supplementary file 1 [file Data_Sheet_1.docx]

**Supplementary Table 1. List of Primers used for quantification of Tocopherol biosynthesis genes**

| Gene ID | Primer sequence (5'->3') |
| --- | --- |
| BnaX.PDS1.a | F: AAGAGGATTGGAGATGTGCTG  R: GTAAACCTTCCCTTCCTCATCC |
| BnaX.VTE1.a | F: CTGAAGAGACCGTTTGAGTAGC  R: TTTTCCTGTATTTGAGCCTCAT |
| BnaX.VTE2.a | F: CTCGCAGTCCGGGCTATTATC  R: GACGTCGCTCCAACTAGAATC |
| BnaX.VTE3.a | F: GGTTCATCCAGCACAAGAAA  R: CCTCTTCCTTTGGTCCAAGCTA |
| BnaX.VTE4.b | F: CATTCAGTCTTCGTTGTGCAAT  R: CCCTTCAATCATCAATGG |
| BnaC.VTE5a | F: AAGAAAGAGCTTGGCGGGAA  R: TCACTAATTGTGGGTTTGGTGC |

**Supplementary Table 2. Interactive effect of genotype, treatment and timings on photosynthetic pigments in rapeseed**

|  |  | **Chl a (mg/g FW)** | **Chl b (mg/g FW)** | **Chla+b (mg/g FW)** |
| --- | --- | --- | --- | --- |
| G x t | G1xt1 | 28.0±3.2 | 16.2±1.3 | 44.2±4.2 |
|  | G1xt2 | 22.2±3.8 | 14.6±1.4 | 36.9±5.1 |
|  | G1xt3 | 20.2±3.4 | 12.9±2.2 | 33.2±5.6 |
|  | G2xt1 | 34.2±2.2 | 22.3±1.4 | 56.6±3.3 |
|  | G2xt2 | 32.0±2.0 | 21.1±1.7 | 53.2±2.8 |
|  | G2xt3 | 30.2±1.6 | 19.9±1.5 | 47.1±3.6 |
| Significance |  | ns | ns | ns |
| T x t | T1x t1 | 36.6±1.5 | 20.6±1.4 | 57.3±2.8 |
|  | T1xt2 | 33.0±1.6 | 19.1±1.9 | 52.2±3.5 |
|  | T1xt3 | 29.9±1.5 | 20.2±1.2 | 50.2±2.5 |
|  | T2xt1 | 25.6±2.2 | 17.9±2.1 | 43.5±4.2 |
|  | T2xt2 | 21.2±3.4 | 16.6±2.3 | 37.9±5.2 |
|  | T2xt3 | 23.3±3.7 | 15.0±2.0 | 35.2±5.6 |
| Significance |  | ns | ns | ns |

G1 = Jiu-Er-13XI, G2 = Zheyou-50), T1= Basal nutrient solution (control), T2 = 100µM Cd, t1= 3 days after treatment (DAT), t2 = 6 DAT, t3 = 9 DAT and ns = non-significant.

**Supplementary Table 3. Interactive effects of genotypes, treatments, and timings on root shoot Cd accumulation and leaf MDA contents in rapeseed**

|  |  | **Cd (root) (mg/g DW)** | **Cd (shoot) (mg/g DW)** | **MDA (nM g^-1^ FW)** |
| --- | --- | --- | --- | --- |
| G x t | G1xt1 | 6.0±2.7 | 0.09±0.02 | 17.4±1.7 |
|  | G1xt2 | 8.7±3.9 | 0.13±0.03 | 22.0±3.0 |
|  | G1xt3 | 11.4±5.1 | 0.16±0.04 | 27.3±3.8 |
|  | G2xt1 | 5.8±2.6 | 0.02±0.001 | 13.8±1.1 |
|  | G2xt2 | 7.1±3.1 | 0.06±0.01 | 16.4±1.5 |
|  | G2xt3 | 8.6±3.9 | 0.08±0.01 | 20.8±1.7 |
| Significance |  | ns | ns | ns |
| T x t | T1x t1 | 0.08±0.01 d | 0.006±0.000 d | 12.8±0.6 |
|  | T1xt2 | 0.09±0.01 d | 0.008±0.001 d | 14.8±1.1 |
|  | T1xt3 | 0.08±0.01 d | 0.005±0.002 d | 20.0±1.3 |
|  | T2xt1 | 12±0.9 c | 0.11±0.03 c | 18.4±1.5 |
|  | T2xt2 | 15±0.9 b | 0.18±0.03 b | 23.6±2.3 |
|  | T2xt3 | 17±3.5 a | 0.23±0.04 a | 29.4±2.8 |
| Significance |  | ** | ** | ns |

G1 = Jiu-Er-13XI, G2 = Zheyou-50), T1= Basal nutrient solution (control), T2 = 100µM Cd, t1= 3 days after treatment (DAT), t2 = 6 DAT, t3 = 9 DAT, ns = non-significant and ** = significant at P≤0.01.

**Supplementary Table 4. Interactive effects of genotypes, treatments, and timings on leaf ROS accumulation in rapeseed**

|  |  | **H_2_O_2_(µmol g^-1^ )** | **O_2_^-^(nmol m^-1^ g^-1^ )** |
| --- | --- | --- | --- |
| G x t | G1xt1 | 34.3±3.0 cd | 56.1±2.4 |
|  | G1xt2 | 44.4±4.5 b | 63.0±3.0 |
|  | G1xt3 | 49.4±5.0 a | 64.3±4.5 |
|  | G2xt1 | 30.4±1.8 d | 50.2±1.9 |
|  | G2xt2 | 32.6±2.0 cd | 56.5±1.5 |
|  | G2xt3 | 36.6±2.0 c | 57.1±2.0 |
| Significance |  | * | ns |
| T x t | T1x t1 | 28.7±1.6 | 49.0±1.4 |
|  | T1xt2 | 31.8±1.5 | 55.4±1.3 |
|  | T1xt3 | 35.8±1.8 | 54.3±1.5 |
|  | T2xt1 | 36.0±2.4 | 57.3±2.0 |
|  | T2xt2 | 45.1±4.3 | 64.0±2.5 |
|  | T2xt3 | 45.3±4.0 | 64.2±3.4 |
| Significance |  | ns | ns |

G1 = Jiu-Er-13XI, G2 = Zheyou-50), T1= Basal nutrient solution (control), T2 = 100µM Cd, t1= 12 hours after treatment (HAT), t2 = 24 HAT, t3 = 48 HAT, ns = non-significant and * = significant at P ≤ 0.05.

**Supplementary Table 5. Interactive effects of genotypes, treatments, and timings on Toc biosynthesis genes**

|  |  | **BnVTE1** | **BnVTE2** | **BnVTE3** | **BnVTE4** | **BnVTE5** | **BnPDS1** |
| --- | --- | --- | --- | --- | --- | --- | --- |
| G x t | G1xt1 | 1.10±0.2 | 0.72±0.1cd | 1.30±0.1b | 1.08±0.05a | 1.5±0.2b | 1.4±0.3d |
|  | G1xt2 | 1.16±0.2 | 0.64±0.1d | 1.19±0.1bc | 0.73±0.04b | 1.5±0.2b | 6.9±2.0b |
|  | G1xt3 | 1.07±0.19 | 1.42±0.1b | 1.64±0.4a | 1.07±0.08a | 1.9±0.1a | 10.8±2.5a |
|  | G2xt1 | 1.20±0.1 | 1.05±0.05bc | 0.93±0.1d | 1.26±0.12a | 0.8±0.1cd | 5.2±2.0bc |
|  | G2xt2 | 1.01±0.2 | 1.32±0.2b | 0.97±0.1cd | 1.13±0.16a | 0.9±0.1c | 4.5±0.3c |
|  | G2xt3 | 1.07±0.1 | 3.58±0.2a | 0.64±0.1e | 1.11±0.25a | 0.6±0.1d | 11.1±3.3a |
| Significance |  | ns | ** | ** | * | ** | ** |
| T x t | T1x t1 | 1±0bc | 1±0cd | 1±0bc | 1±0b | 1±0c | 1±0d |
|  | T1xt2 | 0.65±.05c | 0.62±0.09d | 0.8±0.1c | 0.8±0.1c | 0.9±0.1c | 3.5±0.6c |
|  | T1xt3 | 1.03±0.1bc | 2.23±0.48b | 0.8±0.1c | 0.8±0.1c | 1.2±0.2b | 4.6±0.5c |
|  | T2xt1 | 1.30±0.1ab | 0.78±0.16d | 1.2±0.2ab | 1.3±0.1a | 1.3±0.3ab | 5.6±1.9bc |
|  | T2xt2 | 1.52±0.1a | 1.34±0.27c | 1.4±0.1a | 1.1±0.1b | 1.5±0.2a | 7.9±1.6b |
|  | T2xt3 | 1.12±0.1b | 3.02±0.35a | 1.4±0.4a | 1.3±0.2a | 1.0±0.3c | 15.3±3.2a |
| Significance |  | ** | ** | ** | * | ** | ** |

G1 = Jiu-Er-13XI, G2 = Zheyou-50), T1= Basal nutrient solution (control), T2 = 100µM Cd, t1= 12 hours after treatment (HAT), t2 = 24 HAT, t3 = 48 HAT, ns = non-significant, * = significant at P ≤ 0.05, and ** = significant at P≤0.01.

**Supplementary Table 6. Interactive effects of genotypes, treatments, and timings on leaf Fatty acid content and composition in rapeseed**

|  |  | **16:0** | **18:0** | **18:1** | **18:2** | **18:3** | **Total** |
| --- | --- | --- | --- | --- | --- | --- | --- |
| G x t | G1xt1 | 3.0±0.06c | 0.8±0.1c | 3.8±0.12a | 0.6±0.1e | 4.8±0.2cd | 16.6±0.8d |
|  | G1xt2 | 3.6±0.1b | 1.5±0.1ab | 3.9±0.12a | 1.6±0.2cd | 4.3±0.7d | 18.9±0.9cd |
|  | G1xt3 | 3.8±0.08b | 1.8±0.08a | 2.4±0.16c | 1.8±0.3c | 5.7±1.5bc | 20.8±1.6c |
|  | G2xt1 | 2.8±0.07c | 1.4±0.1b | 3.1±0.03b | 1.4±0.1d | 5.6±0.2cd | 18.6±0.6cd |
|  | G2xt2 | 3.0±0.06c | 1.5±0.1ab | 2.7±0.06bc | 2.7±0.1b | 6.9±0.4b | 23.5±0.7b |
|  | G2xt3 | 4.2±0.1a | 1.6±0.08ab | 2.7±0.15bc | 3.2±0.2a | 9.1±0.3a | 27.1±0.5a |
| Significance |  | ** | * | ** | ** | ** | ** |
| T x t | T1x t1 | 2.8±0.04 | 0.9±0.1 | 3.4±0.2 | 1.0±0.2 | 4.6±0.6bc | 18.8±0.2 |
|  | T1xt2 | 3.2±0.1 | 1.4±0.1 | 3.2±0.3 | 2.4±0.1 | 5.9±0.2b | 21.6±0.3 |
|  | T1xt3 | 3.9±0.2 | 1.6±0.09 | 2.5±0.2 | 2.6±0.1 | 8.6±0.5a | 25.1±0.8 |
|  | T2xt1 | 3.0±0.08 | 1.3±0.2 | 3.5±0.2 | 1.0±0.2 | 4.8±0.2bc | 16.5±0.9 |
|  | T2xt2 | 3.4±0.1 | 1.6±0.06 | 3.5±0.3 | 1.9±0.4 | 5.0±1.2bc | 20.8±1.8 |
|  | T2xt3 | 4.0±0.1 | 1.8±0.08 | 2.5±0.2 | 2.7±0.4 | 6.9±1.3b | 24.1±2.2 |
| Significance |  | ns | ns | ns | ns | * | ns |

G1 = Jiu-Er-13XI, G2 = Zheyou-50), T1= Basal nutrient solution (control), T2 = 100µM Cd, t1= 3 days after treatment (DAT), t2 = 6 DAT, t3 = 9 DAT, ns = non-significant, * = significant at P ≤ 0.05, and ** = significant at P≤0.01.

**Supplementary Table 7. Interactive effects of genotypes, treatments, and timings on leaf tocopherol content and composition in rapeseed**

|  |  | **alpha** | **gamma** | **Total Tocs** |
| --- | --- | --- | --- | --- |
| G x t | G1xt1 | 2.4±0.1 e | 0.10±0.01 b | 2.5±0.1 |
|  | G1xt2 | 2.8±0.1 de | 0.08±0.007 cd | 2.9±0.1 |
|  | G1xt3 | 3.1±0.2 d | 0.06±0.005 d | 3.7±0.4 |
|  | G2xt1 | 3.4±0.1 c | 0.13±0.01 a | 3.6±0.1 |
|  | G2xt2 | 4.3±0.1 b | 0.09±0.009 | 4.4±0.1 |
|  | G2xt3 | 5.1±0.3 a | 0.06±0.005 d | 5.1±0.3 |
| Significance |  | ** | * | ns |
| T x t | T1x t1 | 2.7±0.2 e | 0.14±0.01 | 2.8±0.2 d |
|  | T1xt2 | 3.3±0.3 cd | 0.10±0.006 | 3.4±0.3 c |
|  | T1xt3 | 3.5±0.4 c | 0.07±0.002 | 3.6±0.4 bc |
|  | T2xt1 | 3.2±0.2 d | 0.09±0.01 | 3.3±0.2 c |
|  | T2xt2 | 3.8±0.3 b | 0.07±0.006 | 3.9±0.3 b |
|  | T2xt3 | 4.8±0.4 a | 0.06±0.005 | 5.1±0.33 a |
| Significance |  | * | ns | ** |

G1 = Jiu-Er-13XI, G2 = Zheyou-50), T1= Basal nutrient solution (control), T2 = 100µM Cd, t1= 3 days after treatment (DAT), t2 = 6 DAT, t3 = 9 DAT, ns = non-significant, * = significant at P ≤ 0.05, and ** = significant at P≤0.01.
